# Structural improvement towards the efficiency of biodigesters in the 21st century: a review of the different designs

**DOI:** 10.3389/fbioe.2026.1721607

**Published:** 2026-02-27

**Authors:** Takalani Nethavhanani, Vhutshilo Nekhubvi, Lutendo Mathomu, Nnditshedzeni Eric Maluta

**Affiliations:** 1 Physics Department, University of Venda, Thohoyandou, South Africa; 2 Biochemistry and Microbiology, University of Venda, Thohoyandou, South Africa; 3 Green Technology Confucious Institute (GTCI), University of Venda, Thohoyandou, South Africa; 4 National Institute for Theoretical and Computational Sciences (NITheCS), Pretoria, South Africa

**Keywords:** biodigesters, biogas production, digester efficiency, digester operation, fixed dome digester, floating drum digesters, structural design, tubular digester

## Abstract

Anaerobic biodigesters play a crucial role in the sustainable development of rural areas, managing waste and generating renewable energy. This review evaluates the development and performance of the fixed dome, floating drum, and tubular biodigesters, viz, shows how design improvements and operational strategies impact their efficiency. The original design of these traditional models was found to be restricted by microbial instability caused by climate fluctuations and operational disturbances. In response, modern designs incorporated specific adaptations, such as thermal control and feedstock optimization. The fixed dome model demonstrated improved durability and performance with solar heating and self-mixing capabilities that increase methane production and volatile solids removal. In contrast, floating drum digesters, which are constantly limited by corrosion and inconsistent yields, have been developed to incorporate plastic protective layers, integrated mixing shafts, and *in-situ* purification to achieve better methane concentrations and improved system efficiency. Affordable and adaptable tubular digesters with modular expansion capabilities, incorporating trench burial and greenhouse enclosures, have been designed to enhance affordability and mitigate the effects of climate change. The modifications increase methane production, process stability, and energy recovery. Biodigester performance and efficiency are fundamentally driven by design. Accordingly, the future adoption of anaerobic biodigesters will depend on locally adaptable and affordable systems supported by practical maintenance frameworks, as well as community awareness and training. Overall, recent design innovations have enabled a shift from climate-sensitive traditional models toward more durable, efficient, and adaptable digesters capable of stabilizing methane yield under variable operating conditions.

## 1 Introduction

The global increase in population and rapid industrialization in recent decades has led to a high demand for energy, which has put pressure on existing fossil fuels, which are not only depleting rapidly but also exacerbating environmental issues by increasing greenhouse gas (GHG) emissions (Faisal et al., 2018; Satyanarayana et al., 2011). In response to this, renewable sources are being explored as a more practical alternative to meet energy needs (Kumar et al., 2021). Anaerobic digestion (AD) is a biological process whereby microorganisms decompose biodegradable organic material in an environment free of oxygen, and has proven to be cleaner, more efficient, and helps reduce GHG emissions compared to fossil fuels (Amo Duodu et al., 2021).

Several studies have shown that biogas produced through AD is an effective method for generating renewable energy and treating waste. This process occurs in four stages: hydrolysis, acidogenesis, acetogenesis, and methanogenesis, inside a sealed system called a biodigester (Obileke et al., 2021). The biogas produced primarily consists of methane (50%–70%) and carbon dioxide (30%–50%), along with small quantities (less than 5%) of gases such as hydrogen sulfide, nitrogen, oxygen, and water vapor (Angelidaki et al., 2018). When integrated through biodigesters, this renewable energy source can help reduce energy poverty, indoor pollution, and promote socioeconomic development, with primary domestic uses including cooking, heating, and lighting (Kabeyi and Olanrewaju, 2022; Semelane, 2019).

Although AD technology has the potential to address energy shortages and environmental problems, its growth and adoption have been slow in developing countries, particularly in Africa, where its full potential is not yet reached (Nape et al., 2019). Countries such as China, Bangladesh, Brazil, Kenya, and Tanzania have adopted biogas technology, primarily at small scale levels. China leads the way, having installed 42 million small scale digesters, followed by India with nearly 5 million. In Africa, although Kenya has over 14,000, Uganda 110,000, and Ethiopia 10,000 digesters, adoption is still limited (Pilloni and Abu Hamed, 2021). Given this context, understanding the reasons behind the underdevelopment of this vital energy subsector, particularly household biodigesters, is essential. Although factors such as the lack of subsidized costs, competition from alternative fuels, and the absence of a supportive regulatory framework are often cited as reasons for low adoption, issues related to low efficiency remain a major underlying challenge. The performance of a biodigester is crucial in determining the overall success of the technology (Pilloni and Abu Hamed, 2021; Kluczek and Gladysz, 2022).

To attract investment and promote biogas as a sustainable energy solution, it is important that the biodigester efficiency is improved. Existing research confirms that the efficiency and performance of the AD process depend on various factors, including operating parameters such as feedstock pH, temperature, hydraulic retention time, carbon nitrogen ratio (C/N), organic loading rate, feeding mechanism, digester design, and maintenance (Semelane, 2019; Baniamerian et al., 2019; Rehman et al., 2019). This paper focuses on how the most common biodigester designs influence the efficiency and performance of the AD process. The goal is to identify the best digester configurations, operational practices, and maintenance approaches that maximize efficiency and are more suitable for rural household settings.

## 2 Evolution of biodigesters

A biodigester is an enclosed structure designed to promote the decomposition of organic waste, producing methane gas (Mutungwazi et al., 2018). In terms of scale, biodigesters typically fall into three categories: (i) small scale digesters, which are 5–10 m^3^ in size and have a capacity of less than 25 kW, used for cooking, lighting, or sanitation in rural areas; (ii) medium scale commercial digesters, with capacities ranging from 25 to 250 kW, meant for heating or electricity generation; and (iii) large scale digesters, with capacities exceeding 250 kW, used directly for heating the reactors or converted into combined power and heat systems fed into the grid (Mutungwazi et al., 2018; Msibi and Kornelius, 2017). Household digesters are categorized as small scale, while farm scale digesters usually fall into the medium scale commercial category (Mutungwazi et al., 2018). However, smaller family farms may use systems that are closer to the upper range of small scale digesters (Nape et al., 2019). While these scale classifications may vary between countries, this study adopts this framework for consistency.

Before exploring various biodigester designs, it is essential to investigate the historical development of biodigesters. Jan Baptista van Helmont, a Flemish chemist and physician, made a breakthrough in 1630 by producing flammable gas through the purification of organic matter. Later, in the late 18th century, Italian physicist Alessandro Volta provided scientific insights into the relationship between the decomposition of organic waste and the production of flammable gas (Obileke et al., 2021; Kasinath et al., 2021). The first wastewater sludge digester was built in Exeter, United Kingdom, and it powered streetlamps as early as 1895. Subsequently, in 1897, biogas from human waste lit the Matinga Leper Asylum in Mumbai, India. The first biogas plant using manure was attempted in Bombay in 1900, but it achieved success only by 1937 (Kasinath et al., 2021). Following World War II, Europe witnessed the development of public biogas supply facilities.

The 1970s marked a significant increase in the adoption of biogas in Asian and Latin American countries (Bond and Templeton, 2011). In North America, anaerobic digestion gained momentum in the 1970s, particularly for farm biogas plants, from 25 in 2000 to 176 in 2011 (Obileke et al., 2021; Kasinath et al., 2021). In 2000, Germany had approximately 850 farm-based digesters, which rose to around 7,800 by 2014. China had an estimated 100,000 biogas plants and 43 million residential digesters in 2014, producing approximately 15 billion m^3^ of biogas (Kasinath et al., 2021). India had approximately 4.75 million farm-sized biogas plants in 2014, out of a potential 12 million that could generate around 10 billion m^3^ of biogas annually (Kasinath et al., 2021).

Nepal’s biogas program is among the most successful globally, with over 330,000 household biogas plants installed (Kasinath et al., 2021). In Africa, efforts by international organizations and foreign agencies have promoted biogas technology (Kasinath et al., 2021). South Africa and Kenya were the first African countries to install a biogas digester in 1950, followed by other nations such as Tanzania, Ethiopia, Ghana, Lesotho, Nigeria, Rwanda, Zimbabwe, Guinea, and Uganda, where biogas digesters began as early as 1975 (Amigun and Von Blottnitz, 2010). Several biodigesters were installed in Burundi, Botswana, Burkina Faso, Côte d’Ivoire, Guinea, Namibia, and Rwanda. By 2009, over 53,617 biogas digesters had been installed across the continent, with South Africa estimated to have around 700 (Tolessa, 2024).

## 3 Materials used to construct biodigesters

Biodigesters are usually constructed using durable materials such as bricks, cement, steel, and reinforced concrete (Pal et al., 2025). Some of the materials used in building biodigesters are listed in Table 1. The choice of materials for household digesters depends on geographical, hydrological, and local conditions, as well as availability (Obileke et al., 2021). Over time, these materials can deteriorate, leading to issues like leaks (Obileke et al., 2020). An ideal material for a biodigester should withstand the corrosive environment of biogas, temperature and pressure fluctuations, moisture exposure, and mechanical stresses from the digestion process. Additionally, it should resist degradation over time to ensure long-term durability and optimal system performance (Obileke et al., 2021).

**TABLE 1:** Selected materials used for the construction of biogas digesters, with their advantages and disadvantages (Adapted from Obileke et al., 2021; Mereu, 2016).

Material for construction	Advantages	Disadvantages	Modifications
Bamboo and wood support	Locally available material	Breaks easily	A support material, reinforced with flax
Bricks, cement, and concrete	Enduring, less maintenance cost	Difficult to clean, occupies more space, gas leaks pressure rises (through pores)	Pre-fired earthen rings, lime concrete, slag concrete, fired clay, reinforced concrete
Neoprene and rubber	Weather-resistant elastic	Expensive, low pressure, and a shorter life span	Reinforced with nylon
Polyvinyl chloride (PVC)	Less weight, easily portable	Short lifespan of plastics	Red mud PVC (mixed with aluminium)
Polyethylene (PE)	Cheaper compared to PVC	-	PE with UV filter
Metal (steel drum)	Produces gas at a constant flow, leak-proof	Corrosion, Heavyweight of the gas holder	-

## 4 Various biodigester designs and systems

**FIGURE 1:**
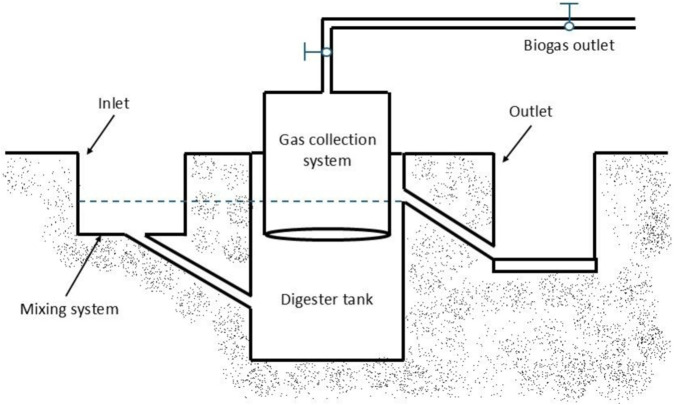
Schematic diagram showing the basic components of a biodigester. Adapted from Rahman et al. (2017).

The structure of a biodigester generally includes several key components designed to support the anaerobic digestion process. Figure 1 illustrates the basic structure of a biodigester, a standard technology used in rural households for lighting, cooking, and heating. These components work together to enable the anaerobic digestion process, converting organic materials into biogas and digestate (Chojnacka and Moustakas, 2024). The main parts of all biogas systems include (i) the reactor/digester tank (chamber): the primary vessel where anaerobic digestion occurs, providing an oxygen-free environment for microorganisms to break down organic matter; (ii) inlet: used to fill the biodigester with organic feedstock, sometimes equipped with a small tank on top (mixing system) that acts as a funnel to simplify filling; (iii) outlet: where the remaining material, called digestate, is collected. Overall, the efficient operation of each component in a biodigester system is crucial for achieving high conversion rates of organic matter to biogas, reducing environmental impacts, and maximizing energy and nutrient recovery (Kabeyi and Olanrewaju, 2022).

To optimize the high performance and efficiency of biodigesters, the different main components as indicated above need to be given high importance during the holistic design and management of biodigesters models (Trois et al., 2021). The three commonly used biodigester models designed for use in rural areas in developing countries are the fixed dome model, the floating drum, and the tubular flow design, which directly reduce the GHG emissions by capturing methane from organic wastes and thus, contributing to climate change mitigation and alternative energy supply (Sahu, 2013). The design of biodigesters has evolved significantly over the years to meet specific applications. They differ according to the climatic conditions, geographic locations, and other factors. Smaller gas volumes tend to work better in mountainous areas as they minimize gas loss, whereas underground digesters are more suitable for utilizing geothermal energy in tropical regions (Rajendran et al., 2012). This section will focus on the structural systems, operation, management, and challenges associated with the three main commonly used models.

### 4.1 Fixed dome biodigester

The fixed dome biodigester was first designed and developed in China, and it is sometimes referred to as a Chinese or hydraulic digester (Rajendran et al., 2012). It features an integrated upper dome and body. It is widely used for biogas production in small scale applications across Sub Saharan Africa (Mungwe et al., 2016). It has an inlet chamber that feeds slurry into the main digester, and the biogas is collected at the top. During the process, the pressure is built up and causes a difference between the digester and the expansion chamber in slurry levels. The pressure and the dome’s constant volume promote the flow of biogas through pipes at the same time pushing some of the slurry into the expansion chamber (Rajendran et al., 2012). To reduce temperature fluctuations during the day and night, these types of digesters are built underground, which also gives an advantage in withstanding the gas pressure on the dome (Mutungwazi et al., 2018).

**FIGURE 2:**
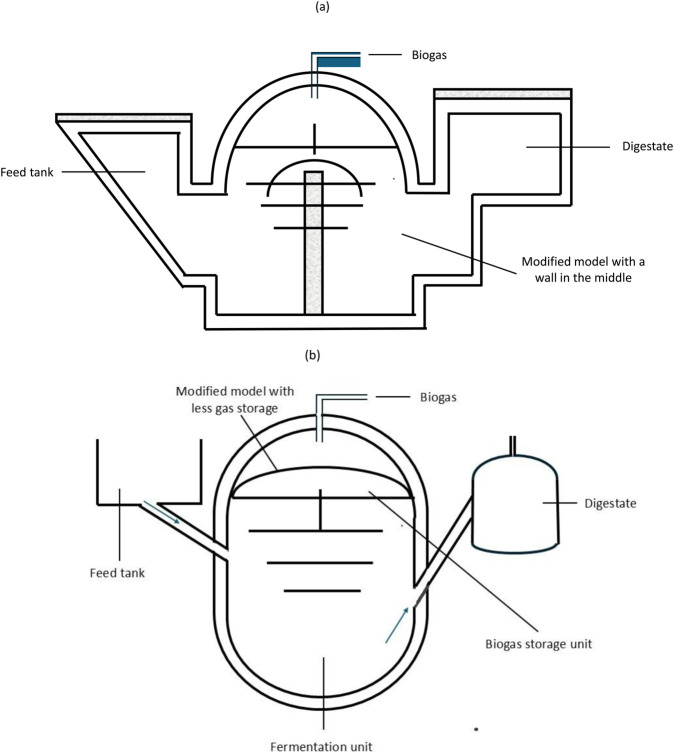
Schematic sketches of fixed-dome biodigesters: **(a)** Janta model and its modifications, and **(b)** Deenbandhu model and its modifications. Adapted from Rajendran et al. (2012).

There are different designs of the fixed dome biodigesters, which mainly differ in shape and share the same basic operation: (i) the Chinese model, created in China and mainly used in rural areas. It has a cylindrical digestion chamber with hemispherical ends; (ii) Janata model: introduced in 1978, it was mostly installed in India and is known for its shallow well with a dome roof. The inlet and outlet are positioned above the dome, with the gas pipe on top. This model allowed substrate recirculation through a partition in the middle, but had issues such as a short slurry circulation path, slurry escaping at the top, and reduced gas production due to high gas pressure. It was later abandoned because of structural problems; (iii) Deenbandhu model, which is a modified version of the Janata design, featuring two spheres of different sizes. The lower sphere functions as the fermentation chamber, and the upper one as storage. The Deenbandhu model was developed to lower costs without sacrificing efficiency, and its shape offers better structural strength (Kabeyi and Olanrewaju, 2022; Rajendran et al., 2012). The Jamata and Deenbandhu models are shown in Figure 2. The brick and mortar fixed dome design usually uses plastic materials to prevent rust and organic matter reactions, lowering construction costs and simplifying the build. The upper part’s fixed dome enhances the design’s durability by eliminating moving parts for gas use. Special gas tight sealants can reduce cracks and porosity caused by pressure between the dome’s parts. While offering benefits, the fixed dome biodigester has significant drawbacks, such as reduced efficiency due to the fluctuating gas pressure (Mutungwazi et al., 2018). The system lacks perturbation to increase the anaerobic reactions, which leads to less production of biogas. Once the gas is in use, the dome cannot maintain the pressure, and the gas supply pressure cannot be maintained. Sometimes the construction materials develop cracks, which delays building processes and necessitates skilled labour. If leaks are detected in the building, it is difficult to fix them, and this can lead to structural failure (Mutungwazi et al., 2018). It has been found that more than half of the structural fixed dome digesters fail within 3 years. In rocky terrains, it is advised not to use the excavation method when designing these types of biodigesters. The poor perturbation of gas causes solid buildup, making maintenance and cleaning more difficult (Mohamed Zaki et al., 2020).

### 4.2 Floating drum biodigester

**FIGURE 3:**
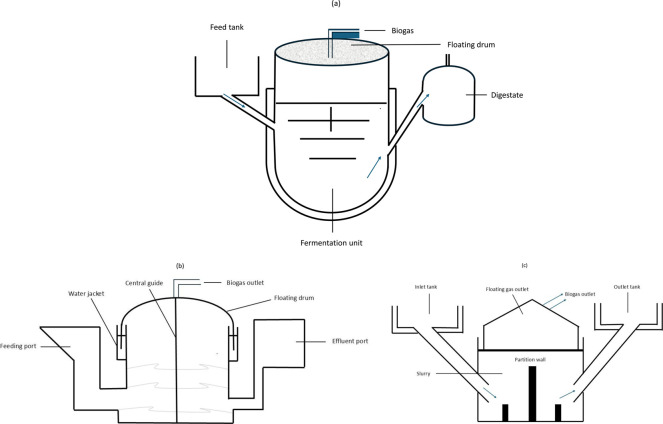
Schematic diagrams of floating drum biodigesters: **(a)** Pragati model, **(b)** KVIC model, and **(c)** Ganesc model. Adapted from Mereu (2016).

First introduced in 1962 by the Khadi and Village Industries Commission (KVIC), this digester has been a reliable solution for biogas generation, especially in household settings (Rajendran et al., 2012; Mereu, 2016). The design is positioned on top of a well-shaped digester and features an oil-paint-coated metal drum that is surrounded by a water jacket consisting of a locking mechanism and a guiding frame for stability purposes. The fermentation chamber functions as the gas holder, providing sufficient space for gas storage at a constant pressure. (Mutungwazi et al., 2018). The floating drum biodigester has three primary models (shown in Figure 3) that operate on the same principle but differ in the shape of the digester chamber: (i) The Pragati model, characterized by its hemispherical chamber, well known for its efficient surface-to-volume ratio, which enhances digestate recirculation; (ii) the KVIC model, identified by its cylindrical chamber with an internal septum that facilitates digestate recirculation; and (iii) the Ganesc model, featuring a conical chamber, offering specific advantages for certain applications. These various biodigester designs are intended to meet different needs within the biogas industry, offering benefits such as ease of operation, a visual gas volume indicator, consistent gas pressure, are leak proof and ensures that minor construction errors that do not significantly impact the gas function and output (Mohamed Zaki et al., 2020; Mereu, 2016; Mutungwazi et al., 2018). Drawbacks related to this technology include regular maintenance costs for the drum coating. Overall, compared to the fixed dome type, the floating drum design is more expensive, and its susceptibility to corrosion reduces its lifespan (Mutungwazi et al., 2018; Rajendran et al., 2012).

### 4.3 Tubular biodigester

**FIGURE 4:**
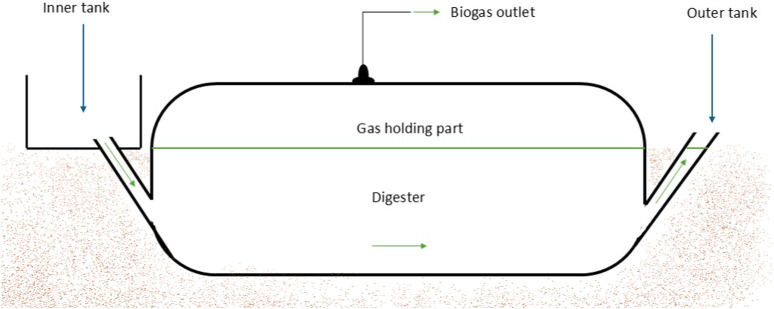
Schematic diagram of the tubular biodigester. Adapted from Mungwe et al. (2021).

The digester types (Tubular or plug flow) were developed, suitable for operation above ground. These installations aim to offer more flexible and adaptable energy solutions for the users, making it easier to relocate and overcome operational issues faced by traditional models (Mutungwazi et al., 2018; Rajendran et al., 2012). The digester that was first installed as plastic tubular digester was installed in Colombia and Ethiopia during the 1980s, and it was later improved in 1992 in Vietnam by replacing the plastic tube with a more affordable polyethylene tube (Mereu, 2016). High quality tubular digesters come as prefabricated bags, with inlet and outlet pipes, eliminating the need for concrete tanks. A basic tubular design is shown in Figure 4. The digester system is placed in a shaped trench with a slight incline from the inlet to the outlet. The biogas is stored in the upper section of the digester, with additional weights placed on the balloon to increase pressure safely. The digester is wrapped around PVC drainpipes and secured with recycled tire rubber tube straps to create an airtight tank for anaerobic digestion. The biogas bags, made from durable reinforced PVC, has safety valves to control gas pressure when weights are added to the balloon (Mohamed Zaki et al., 2020; Mereu, 2016). These models are valued for their simple installation, ease of handling, ability to operate in harsh conditions and for their low cost effective option due to their low skilled labour needs (Rajendran et al., 2012; Mereu, 2016). In mountainous areas, transportation costs for construction materials are high which raises capital expenses and thus building underground large volume digesters at high altitudes very challenging. The plastics that are used to construct biogas bags are susceptible to deterioration from extreme weather conditions, which can lead to blockages due to the buildup of solids, necessitating the use of a pump for gas extraction. The lifespan of the tubular digester is relatively short. The digester faces mechanical challenges, with materials often not available locally and low gas pressure needing extra weights. (Mutungwazi et al., 2018; Rajendran et al., 2012). Common challenges with biogas bags setups include loose clamping of plastics on pipes, gas leaks through uncovered pipes, and difficulty maintaining proper slurry levels due to improper sizing. Exposed digesters are vulnerable to damage from stones and twigs thrown by children, and heavy rain can cause over-dilution of slurry. The absence of agitation leads to scum formation and clogging, worsened by grass and shrubs growing inside the digester due to the earth floor, which promotes plant growth during rainy seasons (Mutungwazi et al., 2018; Rajendran et al., 2012).

## 5 Comparison of the different biodigester designs

At a household level, a biodigester should be simple, reliable, and affordable while effectively addressing the specific needs and challenges of that household. Therefore, to choose the most suitable biodigester design that meets these criteria, it is helpful to compare fixed dome, floating drum, and tubular biodigesters based on various factors such as (i) initial cost; (ii) scalability; (iii) lifespan; and (iv) performance (Mereu, 2016; Amigun and Von Blottnitz, 2010).

### 5.1 Initial investment cost

The initial investment costs usually cover expenses related to the design, materials, construction, and installation of the biodigester system. These costs can vary significantly depending on factors such as location, digester size, system complexity, material quality, labour costs, and any site preparation required (Renwick et al., 2007). The variation in region was seen in a study by Amigun and Von Blottnitz (2010), who showed that the total construction cost for a 6 m^3^ fixed dome digester installed in South Africa’s coastal region was about 1150 USD (7,585 ZAR), which is roughly 34% higher than a similar plant in Rwanda’s landlocked region, costing around 860 US (5,673 ZAR)^1^. Table 2 compares the initial investment costs of the three common designs using a 6 m^3^ household scale unit as the basis for comparison. It’s evident that the fixed dome and floating drum digesters are associated with higher initial investment costs than tubular digesters. This is consistent with Rajendran et al. (2012) and Mungwe et al. (2021), who note that fixed structure biodigesters require relatively high capital investment due to the need for permanent construction materials and skilled workmanship. When compared to the other two designs, the tubular biodigester offers a low investment cost because it does not require specialized labour, is portable, and comes pre-equipped (Martí-Herrero et al., 2014).

**TABLE 2:** Comparison of the main cost factors for different biodigester designs (Adapted from Mereu, 2016; Geddafa et al., 2023; Martí-Herrero et al., 2014; Renwick et al., 2007).

Biodigester design	Main cost factor		Indicative initial investment cost (range)^a^	Qualitative investment cost category^b^
	Skills of contractors needed	Material		
Fixed dome	masonry and plumbing	Bricks and concrete	∼570–859 USD (7,700–11,600 ZAR)	Quite High
Floating drum	masonry, plumbing and welding	Steel drum, bricks, and concrete	∼370–471 USD (3,039–4,722 ZAR)	High
Tubular^c^	plumbing	PVC digester bag	∼320–380 USD (4,310–5,118 ZAR)	Low

^a^ Indicative initial investment cost ranges represent literature-reported values for 6 m^3^ small (household) scale digesters across selected Sub-Saharan African countries. Investment costs generally increase with reactor volume/capacity. The actual costs vary substantially with region, local material/labour prices, subsidy level, and program context. ETB, values were converted using the exchange rate reported in the source study (National Bank of Ethiopia, 27 January 2017): 1 USD, 22.46 ETB. USD, values were converted to ZAR using the corresponding exchange rate for the same date: 1 USD, 13.4688 ZAR.

^b^ The qualitative cost descriptors (“low,” “high,” and “quite high”) are used for comparative purposes only. “Low” indicates the lowest relative capital cost among the three designs, “high” indicates a higher capital cost, and “quite high” indicates the highest relative capital cost for household scale installations.

^c^ Note: For comparison purposes, an approximately 11 m^3^ tubular digester is treated as equivalent in service performance to a 6 m^3^ fixed dome digester.

### 5.2 Scalability

The need for general assessments of scalability play an important role during the selection and design of the biodigester. This process offers flexibility and system adaptability while optimizing resource use and reducing maintenance (Meegoda et al., 2018; Issahaku et al., 2024). Tubular biodigesters have been reported to be a better option for household applications (Mereu, 2016; Pilloni and Abu Hamed, 2021). This is because it can be easily scaled up or down by increasing or reducing tubular modules. The system can be adjusted based on the rates and generation of waste, making it to be suitable for a wide range of household sizes, from small households to larger community level and industrial installations (Mereu, 2016).

### 5.3 Life span of biodigesters

The life span of a biodigester depends on external factors such as climate conditions and the level of regular maintenance it receives. Climate regions have specific characteristics that influence the selection and performance of a biodigester, as these technologies operate best within mesophilic (30 °C–40 °C) and thermophilic (40 °C–55 °C) temperature ranges (Nie et al., 2021). To make a biodigester suitable for cooler climates, it should include insulation and heating accessories (Njuguna Matheri et al., 2018). A study by Leigh (2022) showed that adding four insulation layers and three solar panels to a Flexigester (tubular biodigester) increased the digester temperature to about 33 °C in Wisconsin, United States, where seasonal temperatures vary from 35 °C in summer to −25 °C in winter (Leigh, 2022). However, this setup nearly doubled the retrofitting cost. Similarly, Su et al. (2022) found that in Xuzhou, China, using a solar greenhouse and quilt insulation on a horizontal fixed digester kept the digestion liquid around 24 °C during winter, significantly warmer than the ambient conditions. By optimizing feed times and controlling the insulation quilt, biogas production increased by 15% and 42%, respectively, with combined strategies yielding a 55% boost (Su et al., 2022). These results emphasize the importance of customizing biodigester designs to local climate conditions to ensure reliable, sustainable performance over the long term.

Regular maintenance is essential for optimal operation of fixed dome, floating drum, and tubular biodigesters. Table 3 details the maintenance frequency for each digester type. The fixed dome design requires minimal upkeep, accounting for only about 2% of the initial investment (Kabeyi and Olanrewaju, 2022; Mungwe et al., 2016). Its fully enclosed structure makes repairs impossible if leaks or damage occur (Mutungwazi et al., 2018). The floating drum digester incurs higher maintenance costs due to steel drum corrosion, necessitating repainting every 1–2 years and replacement every 5 years at a high expense (Mutungwazi et al., 2018). The tubular model, on the other hand, is simpler to maintain, involving leak repairs via PVC welding and bag replacement at the end of its lifespan (Mereu, 2016; Martí-Herrero et al., 2014).

**TABLE 3:** Maintenance frequency required for the fixed dome, floating drum, and tubular digesters (Adapted from Mereu, 2016).

Digester design	Maintenance operation	Frequency
Fixed dome digester	None	-
Floating drum digester	Drum painting	Every 1–2 years
	Substitute the steel drum	Every 5 years
Tubular digester	Check PVC bag condition	Every week
	Substitute PVC bag	Every 3–7 years

**FIGURE 5:**
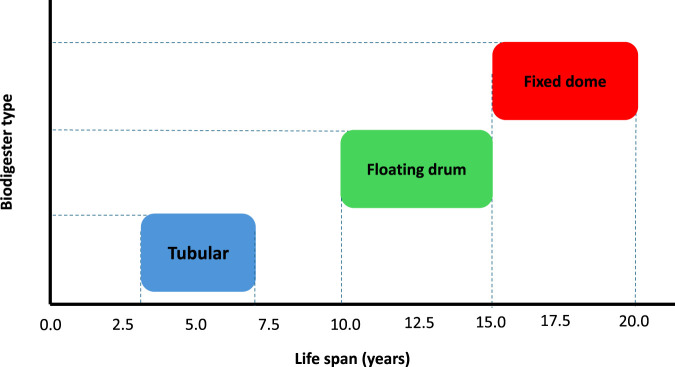
Comparative life span of tubular, floating drum, and fixed-dome biodigester designs. Adapted from Mereu (2016).

To achieve the maximum operational lifetime of biodigesters, the systems must be constructed with durable materials. Poor maintenance and exposure to harsh climatic environmental conditions significantly shorten their service lifespan. Figure 5 illustrates the life of the three main commonly used models, with noticeable differences. It can be noted from the figure that the tubular digesters are the least durable, with an expected lifespan of 3–7 years. This can be related to the construction of polyethylene and related plastic materials, which degrade rapidly under different conditions like rainfall and sunlight (Martí-Herrero et al., 2014). Thus, even when these systems are affordable and easy to install, they are suitable for short term applications due to their limited maintenance and service life. The Floating drum digesters, which fall in the intermediate range, have a lifespan of 10–15 years, depending on the level of maintenance. Their performance is affected by the formation of corrosion on the steel drum, but with protective coatings, their operational life can be extended (Ojo and Ojo, 2023). The fixed dome digesters are durable, with lifespans ranging from 15 to over 20 years. Their reinforced concrete or masonry structure makes them resistant to harsh environmental conditions and relatively easy to repair locally if cracks or leaks occur (Mungwe et al., 2016).

### 5.4 Biodigester performance

Mungwe et al. (2016) assessed how biodigester design and operating conditions influence performance indicators, as they are crucial in determining whether the investment and maintenance costs are justified. A study by Eze and Agbo (2010) investigated a 10 m^3^ masonry biodigester using cow dung as substrate. The biogas observed yielded 0.05 m^3^ for over 20 days. The daily gas production was sufficient to ensure that cooking takes place for 2 weeks. The Nepali fixed dome model treating the mixture of cow and pig manure, where the TS was approximately 16% at HRT of 147 days, produced an average of (1.2 ± 0.8) m^3^/day (Mungwe et al., 2016). The authors, Mungwe et al. (2016), highlighted that due to water scarcity, they were forced to use a less optimal mixing ratio of 3:1 and recommended that regions experiencing water scarcity need alternative configurations that improve mixing. A study by Castaño et al. (2014) used a 1.14 m^3^ modified Chinese fixed dome biodigester fitted with a polyethylene tank, insulated with polyurethane foam, and placed inside a greenhouse for protection from rain and wind, operated at variable temperatures (5.3 °C–27.9 °C) for 363 days to treat dairy manure (VS approximately 6%) produced methane yields of 0.168 m^3^/kg VS added at temperatures above 20 °C. They concluded that maintaining the digester temperature above 20 °C through heating is necessary to ensure stable performance.

Makamure et al. (2024) compared two underground fixed dome digesters: one fitted with a solar heating system (comprising a central heat exchanger, Arduino-based control, and intermittent stirring), and the other with an identical stirrer but no heating system. Both digesters were batch-fed with cow dung and maintained at an average temperature of 35 °C and 25 °C for the heated and unheated systems, respectively, over a 30-day retention time. The temperature controlled and stirred digesters showed improved methane content. Gangagni Rao et al. (2013) compared the performance of a conventional fixed dome (CFD) to a self-mixing dome (SMD)for treating poultry slurry at (37 ± 1) °C. The two digesters, each with a capacity of 2 m^3^, were made of high-density polyethylene (HDPE). The results showed that the SMD outperformed the CFD by tolerating higher loadings. This study highlights that combining a novel biodigester design with intermittent mixing is essential for achieving higher methane yields. Strubbe et al. (2024) evaluated biogas production from a 4 m^3^ household fixed dome biodigester owned by an average family of six members. The digester was fed with cow manure and operated at a retention time of 40 days at an average temperature of 19 °C. Their study revealed that 0.38 m^3^ of biogas per day was produced, meeting about 65% of the family’s cooking needs. These findings reaffirm the vital role of household scale biodigesters in providing renewable energy, among other benefits. Table 4 compares the performance of various fixed dome biodigesters to illustrate how design and operating conditions impact biodigester performance.

**TABLE 4:** Comparative performance of fixed dome biodigesters under different designs and operating conditions.

Digester type and size	Digester material and design	Feedstock	TS (%)	VS (%)	pH	Temp. range (°C)	Retention time (days)	Biogas yield	Methane yield	Methane content (%)	VS removal efficiency (%)	Reference
Fixed dome, 10 m^3^	Masonry	Cow dung slurry	19.8	6.5	∼7.8	35–42	20	0.05 m^3^/day	–	-	–	Eze and Agbo (2010)
Nepali GGC2047 fixed dome, 10 m^3^	Masonry	Variety (pig, cow, fowl, rabbit, goat and garden weed)	16	–	-	18–28	147	1.2m^3^/day	–	-	-	Mungwe et al. (2016)
Chinese fixed dome, 1.14 m^3^	Polyethylene tank, insulate with polyurethane foam, greenhouse cover	Dairy manure slurry	–	6	∼ 7.3	5.3–27.9	363	–	0.176 m^3^/kg VS	–	∼35	Castaño et al. (2014)
Fixed dome (size NR)	Solar-heated, fitted with a heat exchanger, stirred	Cow dung slurry	19.59	15.61	6.4–7.69	35 ± 0.5	30	26.77 m^3^	–	52 -77	76.81	Makamure et al. (2024)
	Stirred	Cow dung slurry	19.59	45.69	6.4–7.69	25	30	18.05 m^3^	–	50 -63	60.37	
Fixed-dome, 2m^3^	Masonry, no mixing	Poultry slurry	2.67	2.15	–	37 ±1	300	0.128 m^3^/kg VS	0.083 m^3^/kg VS	–	42	Gangagni Rao et al. (2013)
Self-mixing dome, 2 m^3^	Intermittent self-mixing	Poultry slurry	5.0	4.0	–	37 ±1	300	0.23 m^3^/kg VS	0.15 m^3^/kg VS	–	64	
Fixed-dome, 4 m^3^	Masonry	Cow manure	–	–	–	19	40	0.38 m^3^/d	–	–	–	Strubbe et al. (2024)

NR-Not recorded/reported.

Studies on floating drum digesters, summarized in Table 5, consistently show that design modifications greatly influence methane yield and gas quality. Sharma et al. (2022) conducted a study in a 6 m^3^ KVIC- type floating drum digester with a calibrated movable gas holder, operating for 60 days under psychrophilic and thermophilic conditions. They used codigested Jatropha de-oiled cake with cattle dung, maintaining TS% at 10%–12%. Their results showed methane concentrations of 62.33%–69.16% and 65.21%–69.15% under mesophilic and psychrophilic conditions, respectively, demonstrating the potential of oilseed residues to enhance biogas quality. Otanocha et al. (2023) highlighted design innovation with a 295 L steel floating drum digester, equipped with an impeller for mixing and integrated purification units (steel wool and activated carbon for H_2_S removal, NaOH scrubber for CO_2_ reduction), achieving nearly 99.8% pure methane. This showed how built-in gas upgrading features can directly improve end-use quality. Their study used cow dung for a retention time of 30 days.

**TABLE 5:** Comparative performance of floating drum biodigesters under different designs and operating conditions.

Digester type and size	Digester material and design	Feedstock	TS (%)	VS (%)	pH	Temp. range (°C)	Retention time (days)	Biogas yield	Methane yield	Methane content (%)	VS removal efficiency (%)	Reference
KVIC floating drum, 6 m^3^	Cemented, gas holder calibrated by uplift height	Jatropha de-oiled cake + cattle dung	JDC:93.2; CD:15.5	JDC:91.5 CD: 83.5	6.82	P: 10–18 M: 22–35	60	P: 0.234 m^3^/kg VS; M: 0.313 m^3^/kg	-	P: 65.53; M: 66.60	P: 23; M: 30	Sharma et al. (2022)
Modified floating drum, 295 L digester	Steel drum incorporated with a cast iron with four impeller shafts	Cow dung	–	–	6.8–7.2	30–40	30	0.003282–0.001969 m^3^/day	–	Up to 99.77 (after purification)	–	Otanocha et al. (2023)
Conventional floating drum reactor, 2.2 m^3^	Movable gas holder	Pretreated wheat straw codigested with cattle manure	-	–	7.2-8.2	34–39	90	0.484 m^3^/kgVS	0.264 m^3^/kgVS	55–56	-	Krishania et al. (2016)
Pilot scale floating dome, 1.2 m^3^	Bottom inlet, flow meter	Potato peel waste slurry (cow dung inoculum)	16.08	92.32	5.3–7.0 (adjusted)	30–35	40	50.27 L/kg	–	-	–	Iyer and Velmurugan, (2024)
Lab scale floating drum, 5 L	Glass cylinder, magnetic stirring	Cow dung + maize waste	-	–	–	36 ± 1	28	230–250 L/kg VS	130–300 L/kg VS	51 - 62	–	Abdoli et al. (2014)

Krishania et al. (2016) discussed limitations of traditional drum geometry when pretreated wheat straw co-digested with cattle manure was fermented in a 2.2 m^3^ floating drum reactor. Their results indicated lower methane yields (0.264 m^3^/kg VS, 55%–57% CH_4_) and biogas yields (0.484 m^3^/kg VS) compared to advanced CSTR and FFR designs. They revealed that continuous stirring in the digester is not always beneficial for methane production. Karne and Bhatkhande (2024) performed anaerobic digestion of model food waste in a 1000 L plastic floating drum digester under mesophilic conditions. Results showed a sevenfold increase in methane yield (0.323 m^3^/kg VS, 77% CH_4_) when nitrogen supplementation optimized the C/N ratio, highlighting the importance of feedstock management and durable plastic construction. Iyer and Velmurugan (2024) used a 1.2 m^3^ lab-scale floating dome digester to treat semi-solid potato peel waste, operating over 40 days. They produced methane concentrations of approximately 46%–50% (∼503 L/kg TS), with performance limited by high feedstock acidity, emphasizing the need for inoculation and buffering strategies. At bench scale, Abdoli et al. (2014) reported that co-digesting cow dung with maize waste in 5 L glass floating drum digesters nearly doubled methane yields (300 L/kg VS, 62% CH_4_) compared to cow dung alone, also reducing hydrolysis time from 11 to 4 days, illustrating how small-scale designs benefit from co-substrates to accelerate digestion kinetics.

Tubular digesters, especially low-cost tubular digesters (LCTDs), have gained significant attention due to their affordability and flexibility. As summarized in Table 6, research consistently indicates that design aspects such as geometry, thermal insulation, and biomass retention mechanisms are crucial for performance. A comprehensive review by Kinyua et al. (2016) found that small-scale tubular digesters typically produce 0.012–0.50 m^3^/kg VS with 21%–76% methane. However, their efficiency largely depends on design features that reduce heat loss and optimize feedstock loading. Ai et al. (2005) used a 2.5 m^3^ stainless steel vertical tubular digester with a vertically oriented packing serving as a biomass carrier and mass transfer enhancer to treat waste activated sludge at 35 °C. They observed that the vertical packing improved microbial retention and mass transfer, resulting in shorter HRTs (∼5 days) and methane yields of 0.26 L/g VS, showing how internal packing design boosts biomass activity and stability. Field-scale studies highlight the importance of thermal protection. Bouallagui et al. (2004) demonstrated in laboratory tests that 18 L tubular digesters treating fruit and vegetable waste were highly sensitive to temperature and hydraulic retention time (HRT). Under mesophilic and thermophilic conditions, biogas yields reached 0.7–1.0 m^3^/kg VS with methane concentrations of 60%–65%. However, psychrophilic operation or shorter HRTs (<12 days) led to acidification and instability, emphasizing the need for temperature management in simple tubular systems.

**TABLE 6:** Comparative performance of tubular biodigesters under different designs and operating conditions.

Digester type and size	Digester material and design	Feedstock	TS (%)	VS (%)	pH	Temp. range (°C)	Retention time (days)	Biogas yield	Methane yield	Methane content (%)	VS removal efficiency (%)	Reference
Vertical tubular digester, 2.5 m^3^	Stainless steel, vertical packing tubes	Waste-activated sludge	∼0.95	∼68	7.0–7.2	35	5	–	0.26 L/g VS	∼60–65	41.7	Ai et al. (2005)
Tubular lab digester, 18 L	Glass, semi-continuous, mechanically mixed	Fruit and vegetable waste	4–10	∼87	6.8–7.6	35	10-20	0.7 m^3^/kg VS	–	∼64	Stable at HRT ≥12 days	Bouallagui et al. (2004)
	Glass, semi-continuous, temp. series	Fruit and vegetable waste	4–10	∼87	7.0–7.8	P:20, M:35, T:55		386–997 L/kg VS (depending on T)	–	56–64	54–87 (higher with T)	Bouallagui et al. (2004)
LCTD, 103 m^3^	Double-layer polyethylene, greenhouse enclosure	Swine slurry	–	–	∼7.6	∼17.7	50	–	0.40 m^3^/kg VS	∼63	77.6	Jaimes-Estévez et al. (2021)
LCTD, 0.75 m^3^	Semi buried, polyethylene/PVC, trench-insulated, solar greenhouse cover	Cattle manure	5.13 ± 0.55	75.05 ± 2.3	∼6.7	∼24.9	∼52	173 L/kg VS	–	62.2-62.76	-	Gaballah et al. (2024)
LCTD, 10 m^3^ and 7.5 m^3^	Polyethylene tubular, greenhouse protected	Cow manure	16.86 ±86	82.20 ± 3.25	7.63 ± 0.46	16.33 ± 1.58	90	0.36 ± 0.04	–	-	-	Garfí et al. (2011)
		Guinea pig manure	27.82 ± 4.96	68.51 ± 5.70	8.79 ± 0.22	19.02 ± 0.82	60	0.03 ± 0.01	-	-	-	
		Cow + guinea pig manure	18.75 ± 3.57	80.18 ± 4.74	7.57 ± 0.84	20.12 ± 0.62	60	0.10 ± 0.03	-	-	-	
Low-cost tubular digester, ∼2 m^3^	Flexible PVC, trench installed	Pig slurry	–	–	6.8–8.2 (avg. 7.7)	28–32	90	0.1–0.4 m^3^/kg VS·d	–	–	∼82	Fouedjio et al. (2025)
Cluster of 3 LCTDs, 5 and 10 m^3^)	Polyethylene, half-buried trench	Domestic wastewater	–	–	–	24 -25	∼126	∼0.555 Nm^3^/day	–	–	-	Jiménez-Paute et al. (2025)

Jaimes-Estévez et al. (2021) investigated the thermal behaviour of a 103 m^3^ greenhouse-covered, double-layer polyethylene digester treating swine slurry. The digester operated stably at psychrophilic temperatures (∼18 °C) for 50 days, producing 63% methane, a specific methane yield of 0.40 m^3^/kg VS, and 77.6% VS removal. They noted that although the plastic tubular digester outperformed other low-cost tubular digesters in biogas output, a greenhouse alone without insulation or black plastic was insufficient to heat the digester. Conversely, Gaballah et al. (2024) found that adding a solar greenhouse and trench insulation to semi-buried tubular digesters increased slurry temperature by 2 °C–3 °C, stabilized pH levels, and improve terms of biogas output, a greenhouse alone, without insulation or black plastic, d solids breakdown, with TS and VS removal reaching up to 55% and 61%, respectively. Similarly, Garfí et al. (2011) reported that 10 m^3^ tubular digesters protected by simple greenhouse structures could sustain biogas production in high-altitude Andean conditions (16 °C–20 °C), demonstrating the effectiveness of low-cost thermal buffering in cold environments. Fouedjio et al. (2025) evaluated the operation of a 2 m^3^ uninsulated, double-layer polyethylene tubular digester treating pig slurry in Cameroon over 90 days. The system maintained an average temperature of 28.8 °C and achieved 82% VS removal with daily biogas yields of 0.1–0.4 m^3^/kg VS. They also recommended additional treatment of the digestate to reduce pathogen levels before using it for food crops. Jiménez-Paute et al. (2025) examined a low-cost wastewater treatment system comprising three sequential low-cost tubular digesters (LCTDs) linked to a granular filtration system (GFS) for domestic wastewater. All three digesters, made from flexible tubular polyethylene geomembrane and partially buried in a trench, demonstrated energy recovery potential with an average biogas production of 0.555 Nm^3^/d. Their operational flexibility allowed them to handle variable flow rates through adjustable hydraulic retention times. Similarly, Quintero et al. (2019) observed that a 9.5 m^3^ tubular digester processing municipal organic waste maintained CH_4_ concentrations of 65.6% and approximately 88% VS removal, aided by stable near neutral pH levels (7.1–7.9).

## 6 Conclusion

The review of the three selected biodigesters shows that design innovations and physicochemical factors significantly influence performance and are important for evaluating biodigester effectiveness. While improvements in design remain the primary driver of biodigester efficiency in the 21st century, fixed dome digesters benefit from features such as burial, larger scale, and the integration of solar heating and self-mixing systems. Floating drum systems have been enhanced through impeller driven mixing, and *in-situ* purification units. Tubular digesters have advanced through low-cost modifications like trench burial, greenhouse enclosures, and passive solar heating. The modifications increase methane production, process stability, and energy recovery. Mesophilic operation consistently yields higher and more stable methane levels, whereas psychrophilic conditions reduce yields and increase the risk of acidification. These findings highlight the importance of aligning digester design with the target operational environment.

Feedstock type and composition are equally important. Codigesting multiple substrates, such as cow dung mixed with maize waste or food residues, improves nutrient balance, enhances microbial activity, and shortens lag phases, resulting in higher methane yields than mono-digestion. Conversely, feedstocks with high acidity or poor nutrient content, like potato peels or nitrogen-deficient slurries, tend to produce less methane and cause instability unless supplemented or balanced. High VS removal (60%–88%) indicates efficient breakdown of organic matter, while methane yields ranging from 0.15 to 0.40 m^3^/kg VS serve as reliable measures of bioenergy recovery. Maintaining a neutral pH (around 7.0–7.5) is associated with optimal microbial activity, whereas deviations caused by acid build-up often lead to system inhibition. The challenges that affect the biodigester’s sustainability, which are mainly water scarcity, enabled users to change feeding ratios from the ideal 1:1 to 3:1 for cow dung in some regions, which reduces the efficiency of the digester. Additionally, inadequate mixing and poor temperature control in low-cost models limit process stability.

Regional contexts, such as Sub-Saharan Africa, often require designs that balance affordability with durability against resource limitations. Beyond energy production, biodigesters offer co-benefits in sanitation and environmental management. Integration with household sanitation systems enhances waste treatment, reduces pathogen loads, and produces safer digestate for use as fertilizer. However, further treatment of effluent is sometimes necessary to reduce microbial contamination to safe levels before applying it to food crops. Overall, these findings demonstrate that while design parameters form the foundation of biodigester performance, their success depends on a combination of operational strategies, feedstock management, physicochemical stability, and adaptability to local conditions. Performance in the 21st century is thus best understood as process-driven yet context-sensitive, requiring tailored innovations that address both technical efficiency and socio-environmental sustainability.

## Footnotes

1 Note: ZAR equivalents were calculated using the exchange-rate approach described by Amigun and von Blottnitz (2010), who converted local costs to US$ using the exchange rate applicable in the year of construction. The USD to ZAR conversion applied here was derived from their Table 4 (South Africa, 2004): R23,000 = US$3,487, giving an implied rate of 1 USD ≈ R6.60.
